# Comparative Study of Two Root Coverage Procedures for Localized Gingival Recessions on Lower Anterior Teeth Using Partially De-Epithelialized Connective Tissue Graft (PE-CTG) Aided by a High-Speed Handpiece: A Retrospective Cohort Study

**DOI:** 10.3390/medicina61020308

**Published:** 2025-02-10

**Authors:** Min-Young Goo, Seung-Kyu Lee, Kyung-Min Kim, Won-Pyo Lee

**Affiliations:** Department of Periodontology, School of Dentistry, Chosun University, Gwangju 61452, Republic of Korea; goo800@hanmail.net (M.-Y.G.); ggyu3866@naver.com (S.-K.L.); kemgmin6@nate.com (K.-M.K.)

**Keywords:** connective tissue, gingival recession, periodontal surgery, root coverage

## Abstract

*Background and Objectives*: Gingival recession is a common periodontal condition that can lead to aesthetic and functional problems if untreated, necessitating the development of effective root coverage techniques. The aim of this study was to compare two different root coverages for localized gingival recession on the lower anterior teeth using a partially de-epithelialized connective tissue graft (PE-CTG). *Materials and Methods*: This study included 18 patients (20 teeth) with lower anterior tooth recession. In the tPECTG group (seven patients, eight teeth), the recipient site was prepared with supraperiosteal tunneling. In the vPECTG group (11 patients, 12 teeth), the recipient site was prepared using the vestibular incision subperiosteal tunnel access technique. In both groups, partially de-epithelialized connective tissue was harvested from the hard palate using a high-speed handpiece diamond burr. The change in root coverage was evaluated based on vertical recession and keratinized tissue (KT) values before surgery and 6 months after surgery. *Results*: The mean root coverage was 89.01% across all cases, with the tPECTG and vPECTG groups achieving 87.85% and 89.78%, respectively. The average KT gain was 3.48 ± 1.37 mm, with the tPECTG group showing 3.94 ± 1.74 mm and the vPECTG group showing 3.17 ± 1.03 mm. No significant differences were found between the two groups for either parameter (*p* > 0.05). *Conclusions*: Within the limitations of this retrospective case series, vPECTG was as effective as tPECTG, but easier. Moreover, in both groups, the keratinized gingival width increased, and the mucogingival junction was maintained.

## 1. Introduction

Gingival recession is a widespread periodontal condition characterized by exposure of the tooth root, often leading to aesthetic concerns, root sensitivity, and an increased risk of root caries [1]. Various surgical techniques have been developed to address this condition, with the primary goal of achieving predictable and aesthetic root coverage [2,3,4]. Among these techniques, the subepithelial connective tissue graft (SCTG) combined with a coronally advanced flap (CAF) has long been regarded as the gold standard due to its predictable outcomes and long-term stability [5].

A previous study reported that MRC for the mandibular anterior region (95.7%) was slightly lower compared to other regions (97.1–100%) [6]. This discrepancy may be attributed to the unique anatomical features of the mandibular anterior teeth, such as a thin gingival phenotype, shallow vestibular depth, crowded teeth, high frenum attachment, and mentalis muscle activity, all of which complicate root coverage and increase the risk of recurrence [7].

Allen proposed a tunneling approach that does not utilize a CAF [8]. However, the exposed portion of the SCTG results in a higher risk of necrosis, reducing the predictability of root coverage. Stimmelmayr et al. addressed this issue using a partially de-epithelialized connective tissue graft (PE-CTG), allowing the epithelial portion of the graft to cover the exposed root surface [9]. This technique eliminates the need for a CAF, maintaining the mucogingival junction and vestibular depth, while reducing the risk of necrosis in the connective tissue.

Stimmelmayr et al. harvested connective tissue while leaving the designed epithelial portion intact using a one-incision technique [9]. This method, however, thins the epithelium, increasing the risk of donor site necrosis. To address this issue, several studies have proposed harvesting grafts as free gingival grafts (FGGs) after de-epithelialization of the intraoral area with a high-speed handpiece and diamond round bur [10,11,12].

Stimmelmayr et al. [9] and Lim et al. [10] utilized the gingival sulcus to create a tunnel for sulcular access at the recipient site. However, this method poses a risk of trauma to the anterior gingival sulcus in the mandible and increases the likelihood of perforation. To address this, the present study prepared the recipient site using the vestibular incision subperiosteal tunnel access (VISTA), as introduced by Zadeh [13]. Traditionally, most mucogingival surgeries are performed via the marginal route. However, apical access approaches [13,14] have recently gained attention due to their ability to reduce the number of incisions, minimize trauma, and provide faster healing. These emerging techniques offer advantages over conventional methods, particularly in challenging anatomical regions, by improving surgical precision and patient outcomes. This study builds on these advances by exploring the efficacy of apical access approaches in root coverage procedures.

Currently, simple and predictable root coverage surgeries for localized gingival recession of the lower anterior teeth are limited. This study retrospectively compared and evaluated two types of PE-CTG performed using a high-speed handpiece bur: tPECTG introduced via tunneling sulcular access, and vPECTG performed using VISTA.

## 2. Materials and Methods

### 2.1. Patient Selection

This retrospective study included healthy patients who visited the periodontal department of Chosun University Dental Hospital between January 2016 and March 2021. The inclusion criteria were as follows: (1) patients with Miller Class I, II, or III localized gingival recession in the lower anterior teeth; (2) those who underwent root coverage using PE-CTG, with all procedures performed by a single experienced periodontologist (W.-P.L.); and (3) those with complete electronic medical records and clinical photographs taken at the 6-month follow-up. The exclusion criteria were as follows: (1) systemic conditions such as uncontrolled diabetes, smoking, or other contraindications for periodontal surgery; (2) insufficient clinical records or missing follow-up data; and (3) severe tooth malposition or active periodontitis. This study was approved by the Institutional Review Board (IRB) of Chosun University Dental Hospital (CUDHIRB-2403-007). All treatment plans and procedures were thoroughly explained to the patients, and written informed consent was obtained before surgery.

### 2.2. Surgical Procedure

All patients underwent comprehensive clinical and radiological evaluations prior to surgery. Gingival recession depth (Rec) and keratinized tissue width (KT) were measured using a calibrated periodontal probe (PGF-W; Osung, Kwangmyung, Korea) at baseline and at the 6-month follow-up. Rec was defined as the distance from the cementoenamel junction (CEJ) to the gingival margin at the deepest point of gingival displacement. KT was measured as the distance from the gingival margin to the mucogingival junction (MGJ), with the MGJ visually identified based on the color differentiation and rolling technique.

#### 2.2.1. tPECTG

**Figure 1:**
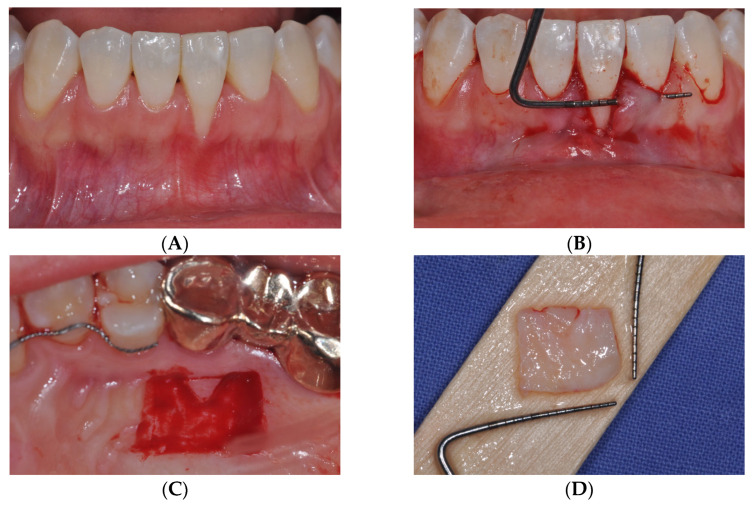

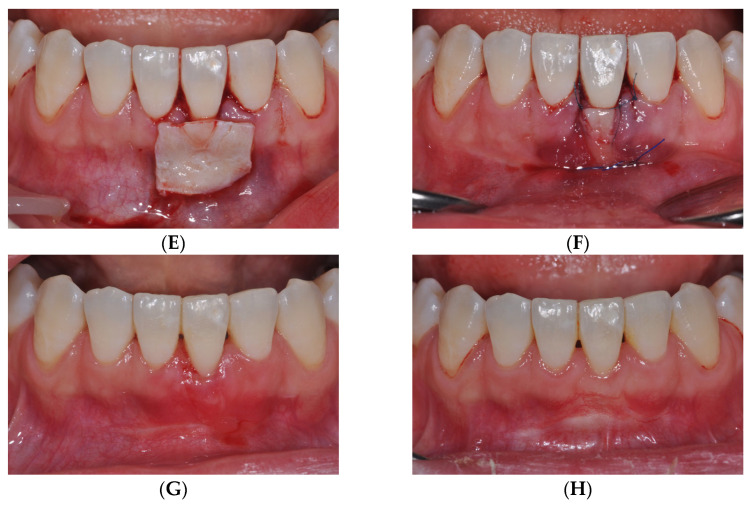
Surgical procedure for tPECTG. (**A**) Clinical view of the recession defects. (**B**) Supra periosteal tunnel preparation. (**C**) Partial de-epithelialization of the graft at the hard palate using a high-speed handpiece with a diamond round bur, leaving the V-shaped epithelialized area intact. (**D**) Prepared partially de-epithelialized connective tissue graft (PE-CTG) with an epithelialized V-shaped region. (**E**) PE-CTG before sutured over defects. (**F**) PE-CTG into tunneling sulcular access and sutured over defects. (**G**) Complete re-epithelialization was noted at 2 weeks post-operation. (**H**) Complete root coverage and keratinized gingiva were obtained 6 months after surgery. tPECTG: PE-CTG with introducing the graft with tunneling sulcular access.

In the tPECTG group, the procedure was performed as previously described [10]. Gargling was performed for 1 min using 0.12% chlorhexidine solution before surgery. After local anesthesia with 2% lidocaine containing epinephrine (1:100,000) (Yuhan, Seoul, Korea), all inflamed tissues were removed using a Gracey curette (LM-Dental, Parainen, Finland), and root planing was performed on the exposed root surfaces. A sulcular incision was made and extended into the adjacent teeth to create a labial supraperiosteal tunnel. At this time, it was ensured that no trauma had occurred to the marginal gingiva. When harvesting the graft from the donor site, it was designed according to the shape and size of the gingival recession defect measured previously from the first to the second premolar area, using a blade. The tissue was de-epithelialized to a depth of 1 mm using a 2-mm diamond round bur and a high-speed handpiece, excluding the part where the epithelium was to be left. Subsequently, a PE-CTG with a thickness of 2 mm was obtained using a blade. The PE-CTG was introduced into the tunnel. The epithelial portion of the graft was then positioned on the exposed root. Suturing and fixation were performed using a non-resorbable monofilament (Rexlon 5–0; Metavision, Seoul, Korea). After suturing, gentle compression was applied to the surgical site for 5 min using a wet gauze. The patient was prescribed ibuprofen 600 mg twice a day for 7 days. The patients were instructed to rinse with 0.12% chlorhexidine twice daily for 2 weeks and to avoid mechanical home care at the surgical site for 6 weeks. The sutures were removed 2 weeks after surgery, and follow-up was performed subsequently (Figure 1).

#### 2.2.2. vPECTG

In the vPECTG group, the procedure for harvesting the donor site was performed in the same manner as that described for the tPECTG group. Gargling was performed for 1 min using a 0.12% chlorhexidine solution before surgery. After administering local anesthesia with 2% lidocaine containing epinephrine (1:100,000) (Yuhan, Seoul, Korea), all inflamed tissues were removed using a Gracey curette (LM-Dental, Parainen, Finland), and root planing was performed on the exposed root surfaces. To form the recipient site, the VISTA method proposed by Zadeh was used to create a labial subperiosteal tunnel while ensuring no trauma to the marginal gingiva [13]. A vertical incision was made in the vestibular region to allow proper access and sufficient tunneling.

When harvesting the graft from the donor site, the tissue was designed based on previously measured gingival recession defect dimensions. A PE-CTG was prepared by partially de-epithelializing the graft to a depth of 1 mm using a 2-mm diamond round bur and a high-speed handpiece. The epithelium on the graft was retained where it would overlay the exposed root. The PE-CTG was introduced into the tunnel and positioned such that the epithelial portion of the graft covered the exposed root surface. Suturing was performed using a non-resorbable monofilament (Rexlon 5–0; Metavision, Seoul, Korea) with a sling-suture technique securing the graft. Additionally, a single interrupted suture was used to close the vertical incision.

**Figure 2:**
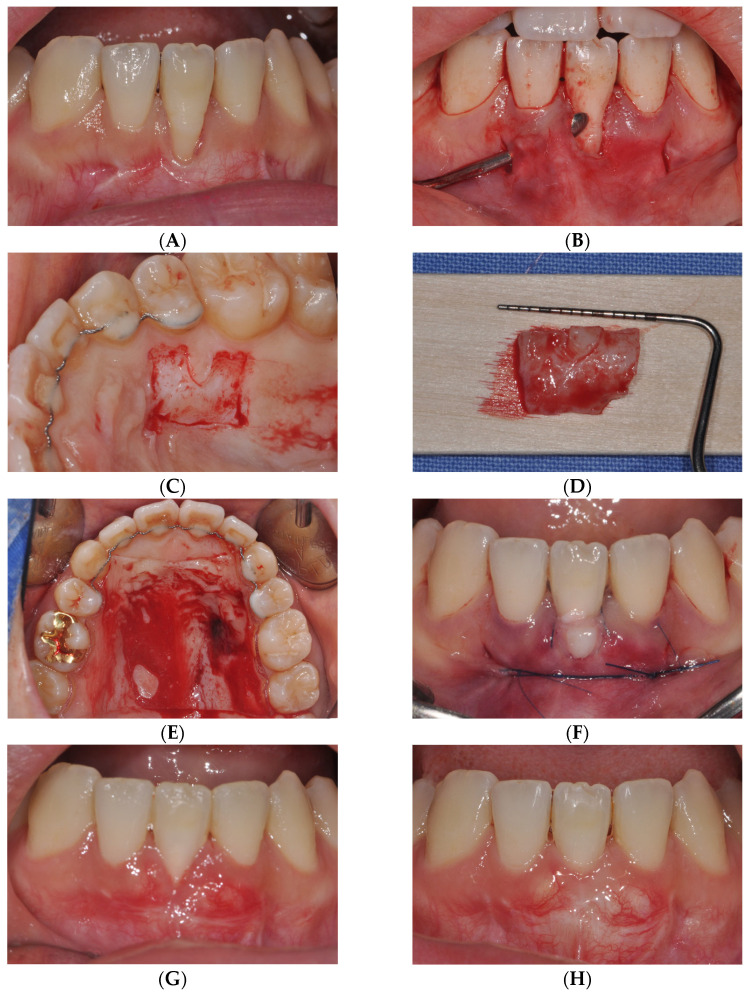
Surgical procedure for vPECTG. (**A**) Clinical view of the recession defects. (**B**) Tunnel preparation with vestibular incision and subperiosteal tunnel access(VISTA). (**C**) De-epithelialization using a high-speed handpiece diamond round bur on the hard palate. (**D**) Partially de-epithelialized connective tissue graft (PE-CTG). (**E**) Wafer application at the donor site. (**F**) PE-CTG into VISTA and sutured over the defects. (**G**) Complete re-epithelialization was noted at 2 weeks post-operation. (**H**) Complete root coverage and keratinized gingiva were obtained 6 months after surgery, which can be attributed to the noticeable effect of creeping attachment. vPECTG: PE-CTG with VISTA technique.

Postoperatively, gentle compression was applied to the surgical site for 5 min using a wet gauze. The patient was prescribed ibuprofen 600 mg twice daily for 7 days and instructed to rinse with 0.12% chlorhexidine twice daily for 2 weeks. Patients were advised to avoid mechanical oral hygiene at the surgical site for 6 weeks. Sutures were removed 2 weeks after surgery, and follow-up care was provided as needed (Figure 2).

### 2.3. Clinical Evaluation

After root coverage was performed on 20 teeth in 18 patients, the percentage of root coverage was evaluated based on the Rec and KT values before and 6 months after surgery.

### 2.4. Statistical Analysis

Each quantitative variable was expressed as an average and standard deviation. The data were tested for normality using the Shapiro–Wilk test. The Mann–Whitney U test was used to test for significance among the groups. The significance level was set at *p* < 0.05. Statistical analyses were performed using SPSS (version 22.0; IBM, Armonk, NY, USA).

## 3. Results

Eighteen patients (20 teeth; 5 men, 13 women) were selected for the study, with ages ranging from 18 to 63 years (mean, 31.8 ± 13.9 years). Among them, Cases 1–8 underwent tPECTG, while Cases 9–20 underwent vPECTG. All cases were diagnosed with Miller Class III and Cairo Type 2 recession. Two patients had two adjacent sites treated, rather than a single tooth. The clinical parameters evaluated are summarized in Table 1, and the comparative clinical results of tPECTG and vPECTG are presented in Table 2.

The overall MRC for both techniques was 89.01%. Between baseline and 6 months after surgery, the Rec change was 3.95 ± 1.32 mm, and the KT gain was 3.48 ± 1.37 mm. In the tPECTG group, the Rec change was 4.25 ± 1.49 mm, while in the vPECTG group, it was 3.75 ± 1.22 mm, with no significant differences between groups (*p* > 0.05). Similarly, the KT change was 3.94 ± 1.74 mm in the tPECTG group and 3.17 ± 1.03 mm in the vPECTG group, also showing no significant differences between groups (*p* > 0.05). However, a statistically significant difference was observed in the baseline width of KT between the two groups (*p* = 0.018), with the vPECTG group showing a higher baseline KT value. The MRC was 87.85% in the tPECTG group and 89.78% in the vPECTG group, with no statistically significant differences (*p* > 0.05). Complete root coverage was achieved in 50% of cases (4/8) in the tPECTG group and 58% of cases (7/12) in the vPECTG group.

## 4. Discussion

This study retrospectively evaluated two novel root coverages in lower anterior teeth. To the best of our knowledge, this is the first study to report the results of a comparison between tPECTG and vPECTG. Moreover, we focused on the clinical and patient benefits of vPECTG, which we recommend for root coverage of the lower anterior region. At 6 months follow-up, the vPECTG group showed similar results to the tPECTG group.

The MRC was 87.9% and 89.8% in the tPECTG and vPECTG groups, respectively, despite all cases being diagnosed with Miller Class III recession. Esteibar et al. [15] reported an MRC of 74.4% in the Miller Class III recession with SCTG introduced by Langer and Langer. Fernández-Jiménez et al. [16] evaluated the modified VISTA technique for treating multiple Miller Class III recessions and observed an MRC of 58.72% after six months, with complete root coverage in 29% of the treated recessions. Additionally, Nart and Valles [17] demonstrated that SCTG combined with a tunnel technique for the treatment of Miller Class II and III gingival recessions in mandibular incisors achieved the MRC of 83.25 ± 14.96%, with statistically significant differences observed between Miller Class II defects (90.92 ± 13.53%) and Miller Class III defects (74.49 ± 11.86%) (*p* = 0.039). Recently, Malhotra et al. [18] evaluated an interdisciplinary periodontal-orthodontic approach versus mucogingival surgery alone for Miller Class III/RT2 recession. Their study reported a significantly higher MRC in the test group (66.67%) compared to the control group (39.93%) (*p* = 0.049). Moreover, Mercado et al. [19] demonstrated that SCTG with enamel matrix derivative yielded superior outcomes in treating multiple Class III-IV recessions in lower anterior teeth. At 36 months, the MRC was significantly higher in the test group (69.85%) compared to the control group (59.29%) (*p* < 0.0007). Compared to these techniques, tPECTG and vPECTG exhibited higher MRC in Miller Class III recessions, suggesting that PE-CTG provides more predictable results than other root coverage procedures for Miller Class III and Cairo Type 2 recessions.

A well-known benefit of root coverage with connective tissue grafts is an increase in keratinized gingiva. Han et al. reported that leaving 1–2 mm of the uncovered graft resulted in a greater increase in KT and prevented displacement of the mucogingival junction [20]. However, when only the connective tissue was exposed, as the amount of graft exposed increased, necrosis of the graft occurred, making complete root coverage difficult. According to Stimmelmayr et al. [9] and Lim et al. [10], when the epithelium is partially left in the connective tissue graft, only the epithelial portion is sloughed off, and vascularization occurs in the connective tissue below. Consequently, predictable root coverage was possible.

Recent studies have reported that primary postoperative pain at the donor site is influenced by factors such as epithelial sloughing during healing and grafting technique [21,22,23]. According to Zucchelli et al., there was no significant difference in the dose of analgesics between the trap-door and FGG harvesting approaches; however, a high dose of analgesics was noted when epithelial sloughing occurred in the trap-door approach [24]. In addition, Harris reported that the graft obtained using the FGG approach was collagen-rich and had little fat tissue [25]. Therefore, partial de-epithelialization with a high-speed handpiece and subsequent harvesting in the form of FGGs, as Lim et al. suggested, can yield collagen-rich connective tissue with low postoperative pain [10].

According to Yotnuengnit et al., the outcome of root coverage is related to the ratio of graft tissue area (GTA) to the visible denuded area (VDA) [26]. They suggested that this ratio should be at least 11:1 to achieve complete root coverage. In this study, the tPECTG group achieved complete root coverage in four of eight cases. The GTA:VDA ratios in these cases were 7.7:1, 10.1:1, 12.4:1, and 7.8:1, ranging from 7.7:1 to 12.4:1. Similarly, the vPECTG group achieved complete root coverage in seven of 12 cases, with GTA:VDA ratios ranging from 5.4:1 to 9.4:1 (ratios observed were 8:1, 9.4:1, 8.9:1, 9:1, 5.4:1, 8.4:1, and 8.5:1). Notably, this study demonstrates that PE-CTG can achieve complete root coverage even with GTA:VDA ratios below the 11:1 threshold proposed by Yotnuengnit et al., indicating the potential of PE-CTG to achieve higher MRC with smaller connective tissue grafts. This finding reduces the graft burden on the patient, which is a significant clinical advantage. Moreover, other factors, such as improved vascularization, graft adaptation, and healing dynamics specific to PE-CTG, may also have contributed to the observed outcomes. These aspects highlight the need for further research to fully understand the factors influencing the success of PE-CTG and its versatility in achieving predictable outcomes in root coverage procedures.

The original technique of CAF combined with SCTG involved the elevation of a partial-thickness flap at the recipient site into the supraperiosteal region to enhance the revascularization of the graft. However, the partial-thickness flap requires more skill than the full-thickness flap, especially in mandibular anterior teeth with a thin phenotype, where the risk of flap perforation is significantly increased. This is particularly true in cases with severe undercuts in the labial apical area, where the gingival sulcus approach further complicates flap elevation. In such situations, vPECTG, which employs the VISTA method and involves full-thickness flap elevation, offers better precision and reduces the likelihood of perforation, making it a preferable approach for thin phenotypes. Mazzocco et al. performed CAF combined with SCTG using full- or partial-thickness flaps and reported no significant difference between the two [27]. These results are consistent with those of this study, where no significant differences were observed in Rec change, KT change, or MRC between tPECTG, which involves partial-thickness flap elevation, and vPECTG, which involves full-thickness flap elevation. Therefore, vPECTG is considered predictive of root coverage in thin phenotypes, such as the mandibular anterior region, where the flap perforation risk is higher.

This study was retrospective in nature and had several limitations. First, the analysis was restricted to cases of localized gingival recession in the lower anterior teeth, which limits the generalizability of the findings to other types of gingival recession. No controls were included to account for variables that could influence the outcomes of root coverage, such as patient age, tooth malposition, and systemic conditions (e.g., smoking and periodontitis). Second, to compare the effects of the two different root coverage techniques, the follow-up period was limited to 6 months to minimize the potential influence of creeping attachment associated with differing observation periods. As a result, the follow-up duration was relatively short, and longer-term follow-up studies are necessary to assess sustained outcomes. Third, the baseline width of KT showed a statistically significant difference between the tPECTG (0.56 ± 0.52 mm) and vPECTG (1.25 ± 0.62 mm) groups (*p* = 0.018), which necessitates careful interpretation of the study’s outcomes. Additionally, the number of subjects in each group was relatively small, and a formal power analysis was not conducted due to the retrospective nature of the study. Nevertheless, despite this limitation, a power analysis based on the current results suggests that approximately 113 teeth per group would be required to achieve statistical significance in Rec with 80% power and a significance level of 0.05. Finally, all procedures were performed by a single experienced periodontist, which, while ensuring consistency in the surgical technique, may limit the generalizability of the results due to the lack of operator variability assessment. We sincerely acknowledge this as a limitation and recommend conducting a prospective clinical study with a robust design, larger sample size, and long-term follow-up to enhance statistical validity and provide stronger evidence for the findings presented here.

Nevertheless, despite these limitations, this study provides valuable insights into the clinical application of root coverage techniques. The findings of this study highlight the effectiveness and practicality of the two root coverage techniques, tPECTG and vPECTG, in addressing localized gingival recession in the lower anterior region. These techniques have demonstrated comparable and predictable outcomes in terms of root coverage and keratinized tissue gain, making them valuable options for treating aesthetically and functionally sensitive areas. The ability of vPECTG to achieve similar results while being less invasive and easier to perform provides a significant advantage, especially in patients with thin phenotypes or challenging anatomical features. Clinicians can leverage these insights to select and customize surgical approaches based on patient-specific needs, ultimately improving patient outcomes and satisfaction. Future research building on these findings will further refine the clinical application of these techniques and validate their long-term efficacy.

## 5. Conclusions

Within the limitations of this study, we concluded that vPECTG was as effective as tPECTG in partially de-epithelialized connective tissue grafts using a high-speed handpiece. In particular, vPECTG is easier to perform and can be performed in thin phenotypes such as those in the mandibular anterior region. In both groups, KT increased, and the mucogingival junction remained unchanged. This is thought to be a valuable treatment for gingival recession in anterior mandibular teeth.

## Figures and Tables

**Table 1:** Baseline and 6-month clinical measures (mm).

				Baseline		6 Months		
Group	Patient	Case	Tooth *	Rec	KT	Rec	KT	% Root Coverage
tPECTG	1	1	#41	6	1	1	5	83.3
tPECTG	2	2	#31	4	0	0	3	100
tPECTG	3	3	#31	4	0.5	0	3	100
tPECTG	4	4	#31	9	0	2	8	77.8
tPECTG	5	5	#41	4	1	0	5	100
tPECTG	6	6	#31	5	0	0	4	100
tPECTG	7	7	#31	4	1	1	4	75
		8	#41	3	1	1	4	66.7
vPECTG	8	9	#41	5	1	0	5	100
vPECTG	9	10	#31	6	1	1	5	83.3
vPECTG	10	11	#41	7	0	1	5	85.7
vPECTG	11	12	#41	3	1	0	3	100
		13	#42	3	1	1	3	66.7
vPECTG	12	14	#31	4	1	0	3	100
vPECTG	13	15	#43	4	2	1	4	75
vPECTG	14	16	#41	4	1	0	5	100
vPECTG	15	17	#41	3	2	1	5	66.7
vPECTG	16	18	#32	3	2	0	5	100
vPECTG	17	19	#31	4	1	0	4	100
vPECTG	18	20	#33	4	2	0	6	100

tPECTG, partially de-epithelialized connective tissue graft with introducing the graft with tunneling sulcular access; vPECTG, partially de-epithelialized connective tissue grafts with vestibular incision subperiosteal tunnel access technique; Rec, vertical recession; KT, keratinized tissue; * FDI tooth numbering system.

**Table 2:** Mean clinical parameters of each group at baseline and 6-month follow-up and mean change in parameters (mm).

	Time	tPECTG (*n* = 8)	vPECTG (*n* = 12)	*p*-Value	Total (*n* = 20)
	Baseline	4.88 ± 1.89	4.17 ± 1.27	0.371	4.45 ± 1.54
Rec	6 months	0.63 ± 0.74	0.42 ± 0.51	0.498	0.50 ± 0.61
	Change	4.25 ± 1.49	3.75 ± 1.22	0.474	3.95 ± 1.32
	Baseline	0.56 ± 0.52	1.25 ± 0.62	0.018 *	0.98 ± 0.66
KT	6 months	4.50 ± 1.61	4.42 ± 1.01	0.887	4.45 ± 1.23
	Change	3.94 ± 1.74	3.17 ± 1.03	0.377	3.48 ± 1.37
MRC		87.85 ± 13.76	89.78 ± 13.73	0.767	89.01 ± 13.42

Rec, vertical recession; KT, keratinized tissue; MRC, mean root coverage; tPECTG, partially de-epithelialized connective tissue graft with introduction of the graft with tunneling sulcular access; vPECTG, partially de-epithelialized connective tissue graft with vestibular incision subperiosteal tunnel access technique. * Statistical significance was established at *p* < 0.05.

## Data Availability

The data presented in this study are available on request from the corresponding author due to ethical restrictions.
